# Angiogenesis Pathway in Kidney Renal Clear Cell Carcinoma and Its Prognostic Value for Cancer Risk Prediction

**DOI:** 10.3389/fmed.2021.731214

**Published:** 2021-10-28

**Authors:** Xiangyu Che, Wenyan Su, Xiaowei Li, Nana Liu, Qifei Wang, Guangzhen Wu

**Affiliations:** ^1^Department of Urology, The First Affiliated Hospital of Dalian Medical University, Dalian, China; ^2^Department of Nephrology, Cheeloo College of Medicine, Shandong Provincial Hospital, Shandong University, Jinan, China; ^3^Department of Breast Surgery, The First Affiliated Hospital of Dalian Medical University, Dalian, China

**Keywords:** kidney renal clear cell carcinoma, angiogenesis pathway, TCGA, bioinformatics, prognosis

## Abstract

Angiogenesis, a process highly regulated by pro-angiogenic and anti-angiogenic factors, is disrupted and dysregulated in cancer. Despite the increased clinical use of angiogenesis inhibitors in cancer therapy, most molecularly targeted drugs have been less effective than expected. Therefore, an in-depth exploration of the angiogenesis pathway is warranted. In this study, the expression of angiogenesis-related genes in various cancers was explored using The Cancer Genome Atlas datasets, whereupon it was found that most of them were protective genes in the patients with kidney renal clear cell carcinoma (KIRC). We divided the samples from the KIRC dataset into three clusters according to the mRNA expression levels of these genes, with the enrichment scores being in the order of Cluster 2 (upregulated expression) > Cluster 3 (normal expression) > Cluster 1 (downregulated expression). The survival curves plotted for the three clusters revealed that the patients in Cluster 2 had the highest overall survival rates. *Via* a sensitivity analysis of the drugs listed on the Genomics of Drug Sensitivity in Cancer database, we generated IC_50_ estimates for 12 commonly used molecularly targeted drugs for KIRC in the three clusters, which can provide a more personalized treatment plan for the patients according to angiogenesis-related gene expression. Subsequently, we investigated the correlation between the angiogenesis pathway and classical cancer-related genes as well as that between the angiogenesis score and immune cell infiltration. Finally, we used the least absolute shrinkage and selection operator (LASSO)–Cox regression analysis to construct a risk score model for predicting the survival of patients with KIRC. According to the areas under the receiver operating characteristic (ROC) curves, this new survival model based on the angiogenesis-related genes had high prognostic prediction value. Our results should provide new avenues for the clinical diagnosis and treatment of patients with KIRC.

## Introduction

Renal cell carcinoma (RCC) ranks as the eighth most diagnosed malignant disease in humans in the United States (1). Kidney renal clear cell carcinoma (KIRC), a highly vascularized tumor that derives from proximal tubular epithelial cells of the nephrons, is to date the most common pathological type of RCC (2, 3). Although the cause of RCC is currently unknown, its incidence may be related to smoking, obesity, occupational exposure to carcinogens (e.g., leather, and asbestos), and genetic factors (e.g., missing tumor suppressor genes) (4). As ~70% of the kidney cancers are localized or locally advanced when first diagnosed, they can be treated *via* surgical resection; therefore, radical nephrectomy is the most common treatment (5). However, approximately one-third of the patients who have undergone renal tumor resection still develop distant metastasis eventually (6). Unfortunately, metastatic RCC is highly resistant to the traditional radio- and chemotherapies. Over the last decade, molecularly targeted drug therapy and immunotherapy have been used clinically to treat the advanced kidney cancer. However, many patients develop drug resistance and eventually experience cancer progression. Such setbacks force us to rethink the current treatments for RCC.

A tumor metastasis is a gradual, multistep process that involves angiogenesis, cell invasion, migration, and adhesion, and extravasation into target organs, where angiogenesis and the other processes are repeated indefinitely (7, 8). The decreased oxygen concentration in the solid tumors leads to the accumulation of hypoxia-inducible factor and increases the expression of pro-angiogenic factors, such as vascular endothelial growth factor (VEGF) and platelet-derived growth factor (PDGF) (9). The studies have shown that the angiogenesis signaling pathway plays an important role in the pathogenesis of many solid tumors, such as RCC (10). As a strategy to deprive tumors of their nutrition and thereby inhibit their growth, the anti-angiogenesis drugs have become the main treatment for human cancers. Tyrosine kinase inhibitors, such as sorafenib and sunitinib, have been used as a first-line treatment for metastatic KIRC (11, 12). However, most of the molecularly targeted therapies have been less effective than expected, as assessed through their associated progression-free survival, objective response rate (ORR), and overall survival (OS) outcomes (13). Moreover, the patients with the same disease stage and similar treatment regimens have shown considerable variations in the clinical outcomes. The recent studies have shown that molecular heterogeneity is involved in the carcinogenesis and development of KIRC tumors, which leads to variation in their cell proliferation, metabolic activity, and tumor microenvironment (14, 15).

The rapid development of bioinformatics and public databases has accelerated the study of many types of cancers by the research community (16). In this study, we used gene data and clinical information from The Cancer Genome Atlas (TCGA) database to analyze the copy number variations (CNVs), single nucleotide variations (SNVs), and expression level changes in the genes related to the angiogenesis signaling pathway in 32 tumors and the relationship between the gene expression in each tumor and patient prognosis. We found that the most angiogenesis-related genes had protective effects in the patients with KIRC. To further explain this phenomenon, we systematically evaluated the angiogenesis pathway genes and prognosis in KIRC using the bioinformatics methods. To this end, we divided the dataset of patients with KIRC into three clusters according to their final angiogenesis score and angiogenesis-related gene expression levels and explored the relationships between these three clusters and patient prognosis as well as those between the clusters and drug sensitivity. Because it has been increasingly demonstrated that the immune cells play a central role in tumors (17, 18), we explored the correlation between the angiogenesis score and immune cell infiltration. Finally, we established a risk score model composed of 15 angiogenesis pathway genes to predict the clinical outcome of patients with KIRC. Our research results can provide new avenues for the clinical diagnosis and treatment of patients.

## Materials and Methods

### Acquisition of Angiogenesis-Related Gene Data and Clinical Data

The 24 angiogenesis-related genes investigated in this study were obtained from the Gene Set Enrichment Analysis (GSEA) package on the WikiPathways website (https://www.gsea-msigdb.org/gsea/index.jsp). The TCGA datasets (https://portal.gdc.cancer.gov) were downloaded to obtain the CNVs, SNVs, and expression level changes in these genes in 32 different types of cancer. The data were analyzed with the Perl language and R Studio, and the results were visualized using Toolbox for Biologists (TBtools) (19). Additionally, RNA-Seq transcriptome data and associated clinical data on the patients with KIRC were obtained from TCGA, which contains 539 tumor samples and 72 normal samples. “Corrgram” in RStudio (MA, USA) was used to plot the co-expression relationships among the angiogenesis-related genes, with Pearson's correlation coefficient applied in the statistical analysis of the results.

### Cluster Analysis Based on Angiogenesis Scores

Due to the large variations in gene expression profiles observed in the previously obtained datasets, we constructed an angiogenesis score model based on mRNA expression to show the differential expression levels between the samples. In brief, a single-sample gene set enrichment analysis (ssGSEA) was first used to evaluate the enrichment scores of genes in the angiogenesis pathway. The “Gplots” package in RStudio was used to perform the differential analysis, and the “pheatmap” package was used to draw the heat map of the cluster analysis results. Through comparisons with the mRNA expression levels of the genes in normal tissues, the mRNA expression statuses in the tumor tissues were classified into three categories: angiogenesis inactive (cluster 1 or C1), angiogenesis active (cluster 2 or C2), and normal angiogenesis (cluster 3 or C3). To further illustrate the relationships between the gene expression levels of these three clusters, we used the violin plot drawn with the “ggpubr” package to depict the enrichment score levels of the clusters. Finally, “pheatmap” in RStudio was used to draw a heat map of the relationship between two of the clusters and the clinicopathological characteristics of the patients with KIRC. The differences with a *p* < 0.05 were considered statistically significant.

### Drug Sensitivity Analysis Based on the Genomics of Drug Sensitivity in Cancer (GDSC) Database

Of the 266 drugs listed in the 2019 edition of the Genomics of Drug Sensitivity in Cancer (GDSC) database (https://www.cancerrxgene.org/), which is the largest of its type on pharmacogenomics, we selected 12 classical and novel targeted drugs used for the KIRC treatment for study. On the basis of the cell expression profiles indicated on the GDSC database, we used the “pRRopheticl” package to construct a ridge regression model to estimate the half-maximal inhibitory concentration (IC_50_) of the drugs in the three clusters (20). We also used 10-fold cross-validation based on the GDSC training set to evaluate the prediction accuracy. Except for “combat” and “allSoldTumours” tissue types, all the parameters were set to default values, and the duplicate gene expression levels were summarized as the mean value. Finally, the “ggplot2” and “cowplot” packages were used to draw the boxplot. The results with a *p* < 0.05 were considered statistically significant.

### Classical Cancer-Related Genes and Histone Modification

To explore the potential regulatory mechanism of the angiogenesis pathway in KIRC, we examined the expression levels of various oncogenes and tumor suppressor genes in the three clusters in the form of a heat map, using the “string,” “pheatmap,” “gplots,” and “gird” packages in RStudio. One-way ANOVA was used to compare the expression levels of various oncogenes and tumor suppressor genes in KIRC in the different clusters. The differences with a *p* < 0.05 were considered statistically significant. Similarly, we used the same approach to demonstrate the differences among the three angiogenesis clusters in terms of the expression of sirtuins (SIRTs) and histone deacetylases (HDACs), which aside from being involved in histone modification also play an important role in regulating the angiogenesis pathway.

### Correlation Between the Angiogenesis Score and Immune Cell Infiltration

A ssGSEA was used to quantify the 29 immune-associated gene sets obtained from TCGA and there are a total of 707 genes, representing different immune cell types, functions, and pathways (21–23). The “ggplot2” and “dplyr” packages in R Studio were then used to draw a heat map of the correlation between the angiogenesis-related genes and immune cell infiltration, with Spearman's correlation coefficient being applied for the statistical analysis. A ssGSEA can be applied to gene signals expressed by the immune cell populations in a single sample. Therefore, on the basis of the ssGSEA results, we used the “ggstatsplot,” “data.table,” “dplyr,” “tidyr,” and “ggplot2” packages in RStudio to analyze and plot the correlation between the angiogenesis score and immune substances. In the plotted figure, the area of each sphere represents the degree of correlation and the color represents the *p*-value. Finally, we used the “ggscatterstats” package to generate a scatter plot for representing the correlations between the four classical immune cell populations (viz., T follicular helper (Tfh) cells, mast cells, neutrophils, and type II interferon (IFN) response cells) and the angiogenesis score. A *p* < 0.05 was considered to be statistically significant.

### Construction of a Risk Model Using the Least Absolute Shrinkage and Selection Operator (LASSO)–Cox Regression Analysis

We used the “corrplot” package to show the co-expression relationship between any two of the angiogenesis pathway genes. The “glmnet” package in RStudio was used to perform a least absolute shrinkage and selection operator (LASSO)–Cox regression analysis to further identify the most useful prognostic genes and to establish a risk model. Next, we computed the risk score (RS) of each sample on the basis of the gene expression and coefficient values, using the following formula:


$$\begin{array}{l} \text{RS=}\sum_{\text{i=1}}^{\text{n}}\text{coefi }\times \;{\text{x}}_{\text{i}} \end{array}$$

where coefi represents the coefficient and x_i_ represents the expression value of each selected gene. We used the “survminer” package to obtain the best cut-off value for dividing the samples into a high-risk group and a low-risk group as well as the “survival” package in R to compute the survival curves of these two groups. A receiver operating characteristic (ROC) curve was generated using the “survival-ROC” package in R to obtain the area under the ROC curve. To highlight the superiority and accuracy of our model, we carefully compared our prognostic model with another three prognostic signatures (an autophagy-related long non-coding RNA signature constructed by Yu et al., an m6A-related lncRNA signature constructed by Yu et al., and a seven-MDEG signature constructed by Hu et al.) (24–26). All the genes used for the construction of prognostic signatures of Yu and HU were obtained. The “timeROC” R package was further applied to compute the area under the curve (AUC) values of each model. Finally, based on this model, we analyzed the correlation of the RS with the clinicopathological characteristics of patients with KIRC and used a heat map to depict it. There were no N-stage data because of the large amount of Nx data for these patients in the dataset of TCGA. A *p* < 0.05 was statistically significant.

### Construction of a Nomogram for Predicting the Outcome of Patients KIRC

The univariate and multivariate Cox regression analyses were used to determine the correlations of the patient age, tumor stage, tumor grade, tumor size (T), and tumor metastasis (M) with the RS in the model. Finally, the “rms” package in RStudio was used to draw a nomogram from the Cox analysis results and clinical characteristics for evaluating the survival probability of patients with KIRC.

### Renal Cancer Cell Line, Plasmid Transfection, and Cell Counting Kit-8 (CCK-8) Assay

The renal cancer cell line 786-O was purchased from the Institute of Cell Research, Chinese Academy of Sciences (China). The cells were routinely cultured in Roswell Park Memorial Institute (RPMI) 1640 medium, containing 10% fetal bovine serum and penicillin-streptomycin, at 37°C under a 5% CO_2_ atmosphere. Once the culture had reached the logarithmic growth phase, 2 × 10^5^ cells were seeded into each well of 6-well plates. On the following day, 100 μl of a mixture containing Lipofectamine 3000 (Invitrogen, Carlsbad, CA, USA) and plasmid fragments carrying the tissue inhibitor of metalloproteinase 3 (*TIMP3*) gene diluted in serum-free medium was added to each well. After 6 h of incubation at 37°C, the medium was changed to serum-containing medium and the plates were further incubated for 24 h. Finally, the cells were digested with trypsin and collected for cell proliferation analysis using the CCK-8 assay (Dojindo, Japan). In brief, 1 × 10^3^ cells were seeded into each well of 96-well culture plates (four replicate wells per group). Subsequently, 10 μl of the CCK-8 reagent was added to each well according to the instructions from the manufacturer, and the plates were incubated in a 37°C incubator for 2 h. Finally, the absorbance of each well was measured at 450 nm using a microplate reader (EL340; BioTek Instruments, Hopkinton, MA, USA).

## Results

### Widespread Genetic Mutations of Angiogenesis Pathway Genes in 32 Types of Cancer

**Figure 1:**
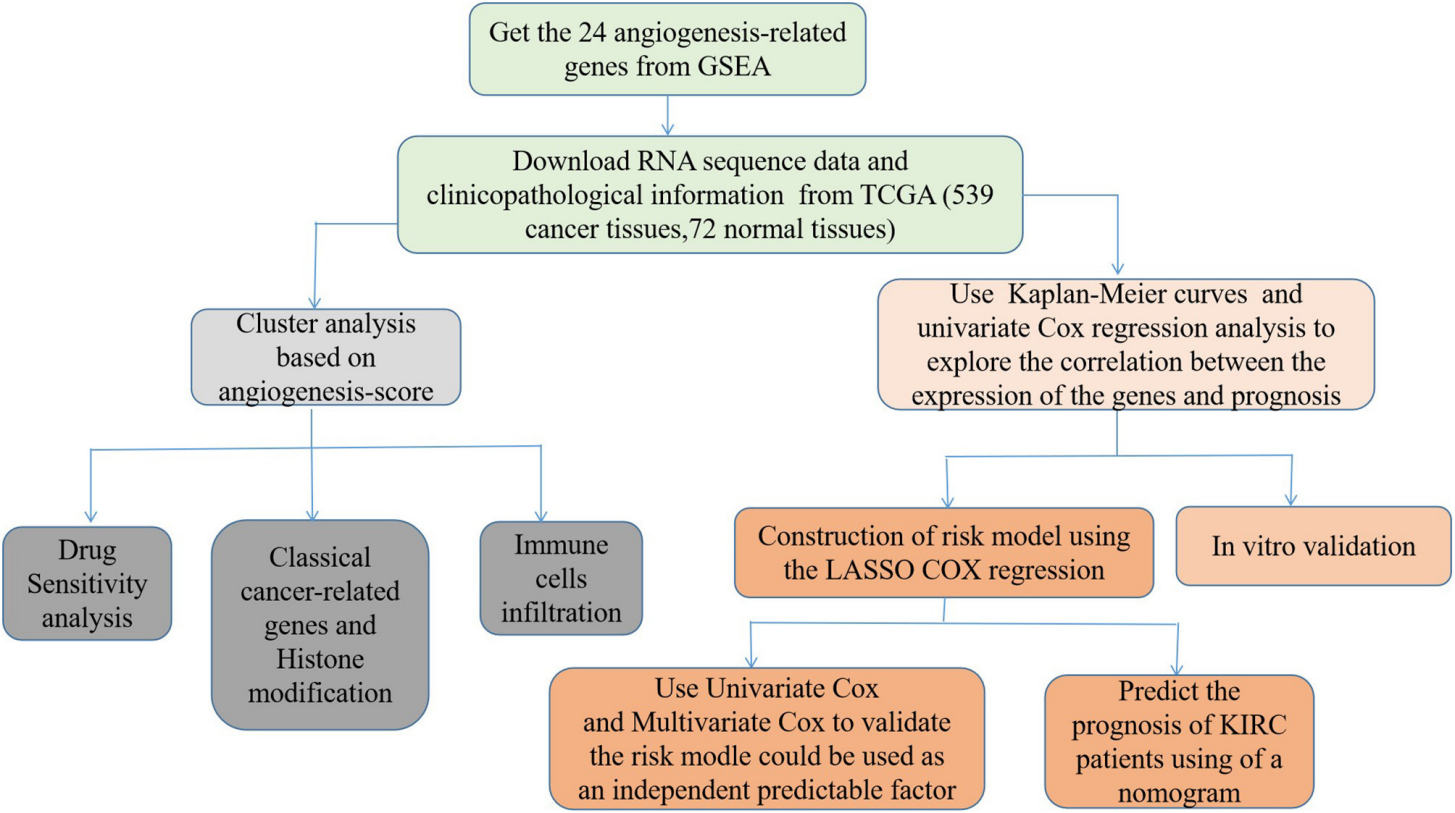
The flowchart of this study.

**Figure 2:**
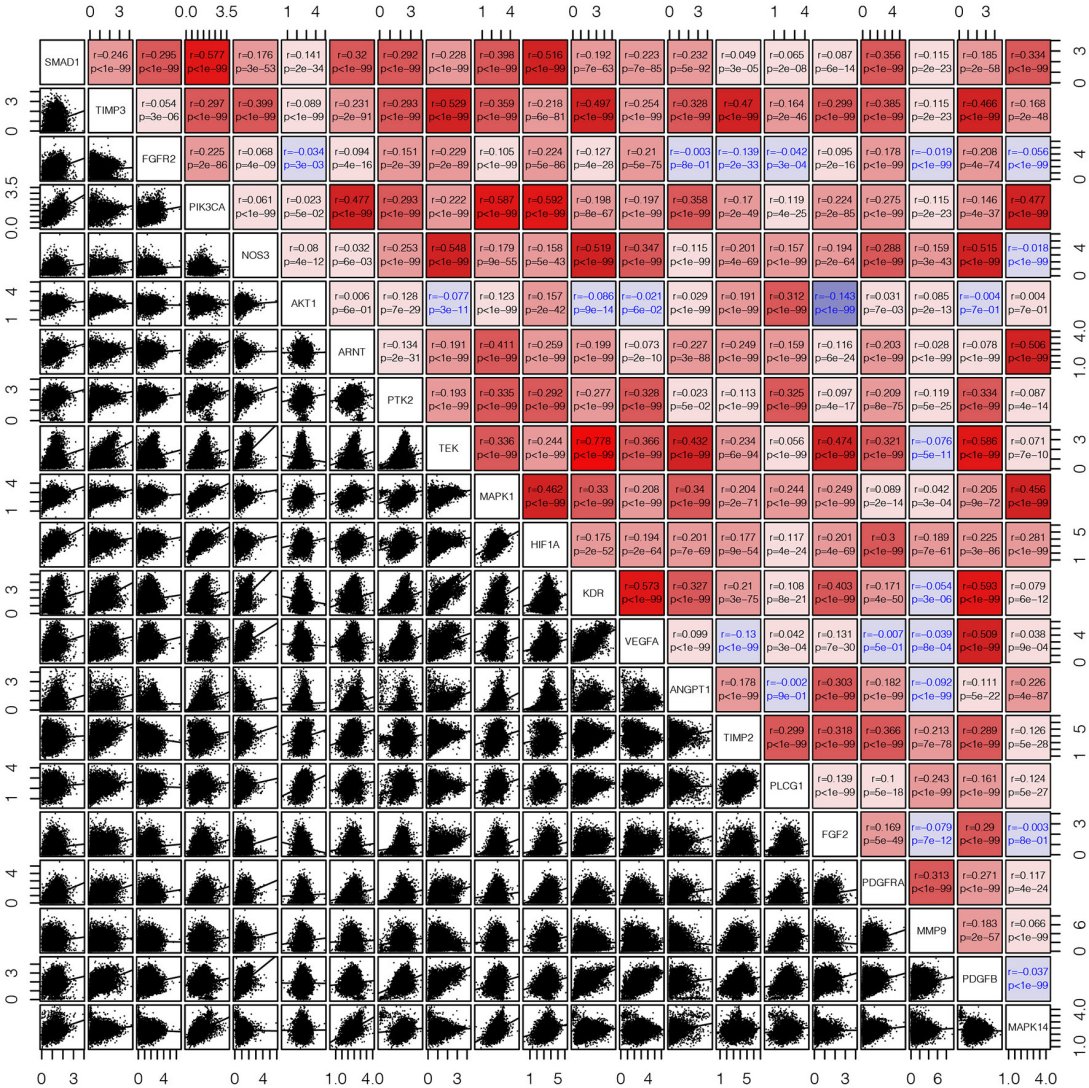
The corrgram showed that there was a co-expression correlation between the angiogenesis pathway genes in pan-cancer. We used the “pair” function in the graphics R package to draw this picture. The gene expression data were normalized by log(1+x). The scatter plot and regression line are drawn in the lower-left panel. The corresponding genes are displayed on the diagonal, and the Pearson's correlation coefficient and the corresponding *p*-values are displayed in the upper right panel. The Pearson's correlation coefficient is projected to five colors, negative values are displayed in blue, and positive values are displayed in red.

First, we draw a corresponding flowchart (Figure 1) to show the process of this whole research more clearly. In total, 24 angiogenesis-related genes were obtained from the GSEA website and referenced to TCGA datasets to obtain their CNVs and SNVs in 32 different types of cancer (Supplementary Figures 1A–C, Supplementary Tables 4–3). Although there were CNVs and SNVs in the angiogenesis pathway genes of most of the cancer types, there were almost no CNV gains or losses in these genes in thyroid cancer (THCA), thymoma, pancreatic adenocarcinoma, KIRC, and prostate adenocarcinoma. There was a high frequency of SNVs in uterine corpus endometrial carcinoma (UCEC), skin cutaneous melanoma, and colon adenocarcinoma. By contrast, the frequency of SNVs was low in kidney chromophobe (KICH), pheochromocytoma and paraganglioma, THCA, and uveal melanoma. Next, we used the expansion package in the R-language program to draw an image showing co-expression between the genes, whereupon we observed that there was a highly positive correlation among the genes *FLT1, PDGFB, VEGFA, KDR*, and *TEK*, whereas there was a negative correlation between *SRC* and *FLT1*. There was also a negative correlation between *SRC* and *KDR* and between *SRC* and *TEK* (Figure 2).

### Roles of Angiogenesis Pathway Genes in Cancer

**Figure 3:**
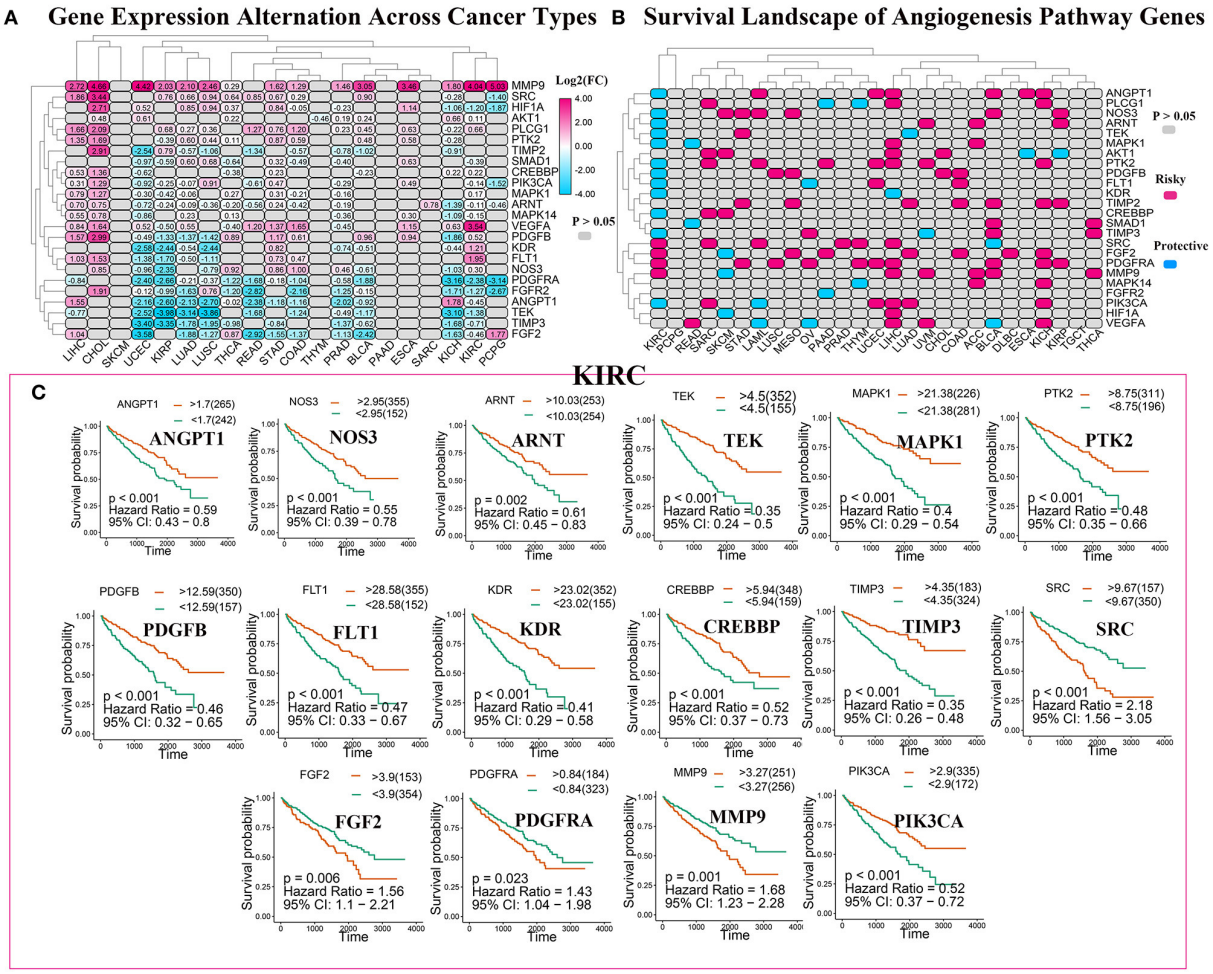
The roles of angiogenesis pathway genes in cancer. **(A)** The changes in the expression of 24 angiogenesis pathway genes among 32 tumor types. The color code bar on the right side shows the corresponding value of log2(FC). And negative changes are displayed in blue, and positive changes are displayed in red. The *P* > 0.05 indicates no statistical significance and is displayed in gray. **(B)** Heatmap showed the survival landscape of angiogenesis pathway genes. Blue represents the protective genes and red represents risky genes. The gray bar represents no statistical significance. **(C)** The survival curve of angiogenesis pathway genes in kidney renal clear cell carcinoma (KIRC). We divided the genes into the high-expression and low-expression groups based on the best cut-off values using the “survminer” package and then plotted the survival curve. The orange line represents the high-expression groups; the green line represents the low-expression groups. The abscissa represents the number of days, and the ordinate represents the survival probability.

To analyze the expression changes in the angiogenesis-related genes in different cancer types, we used log2(fold change) to represent the ratio of the gene expression levels in the cancer tissues to those in the corresponding normal tissues. We found that most of the gene expression levels in the cancer tissues were different from those in the normal tissues (Figure 3A, Supplementary Table 4). Next, we constructed a survival landscape of these genes according to the correlation between the patient survival rate and gene expression in TCGA (Figure 3B, Supplementary Table 5). The gene is regarded as a protective gene when the hazard ratio (HR) is <1 and as a risk gene when the value is >1. Since the angiogenesis pathway itself is a cancer-related pathway, the angiogenesis-related genes were present as risk genes in most cancer types. Usually, the protective genes are downregulated and the risk genes are upregulated in tumors. Interestingly, we found five genes that were all risk factors in UCEC but were downregulated, which seemed contradictory. The following could be possible reasons: (1) tumors are heterogeneous, and the same genes are expressed differently in different tumors. (2) These risk genes are not the drivers of tumors and cannot directly lead to their occurrence and onset, and their expression may be regulated by other genes. (3) What we analyzed above was the mRNA expression of related genes; however, these genes ultimately play roles through their encoded proteins. Sometimes, the post-translational modifications, epigenetics, negative feedback, and other factors may lead to the mRNA and protein expression levels being inconsistent. We also observed from the pan-cancer analysis that most angiogenesis-related genes were protective genes in the patients with KIRC, an interesting phenomenon that was not evident in many other cancers, such as UCEC and KICH. Considering that an abundant blood supply, vigorous lipid metabolism, and insensitivity to radiotherapy and chemotherapy are all characteristics of KIRC, we focused on the relationship between the angiogenesis pathway genes and this disease in the following study. Using the “survminer” package, we divided the genes into the high-expression and low-expression groups based on the best cut-off values. Subsequently, we plotted Kaplan–Meier curves for identifying the statistically significant angiogenesis pathway genes in the patients with KIRC (Figure 3C). The results were consistent with the constructed survival landscape (Figure 3B).

### Cluster Analysis Based on the Angiogenesis Scores

**Figure 4:**
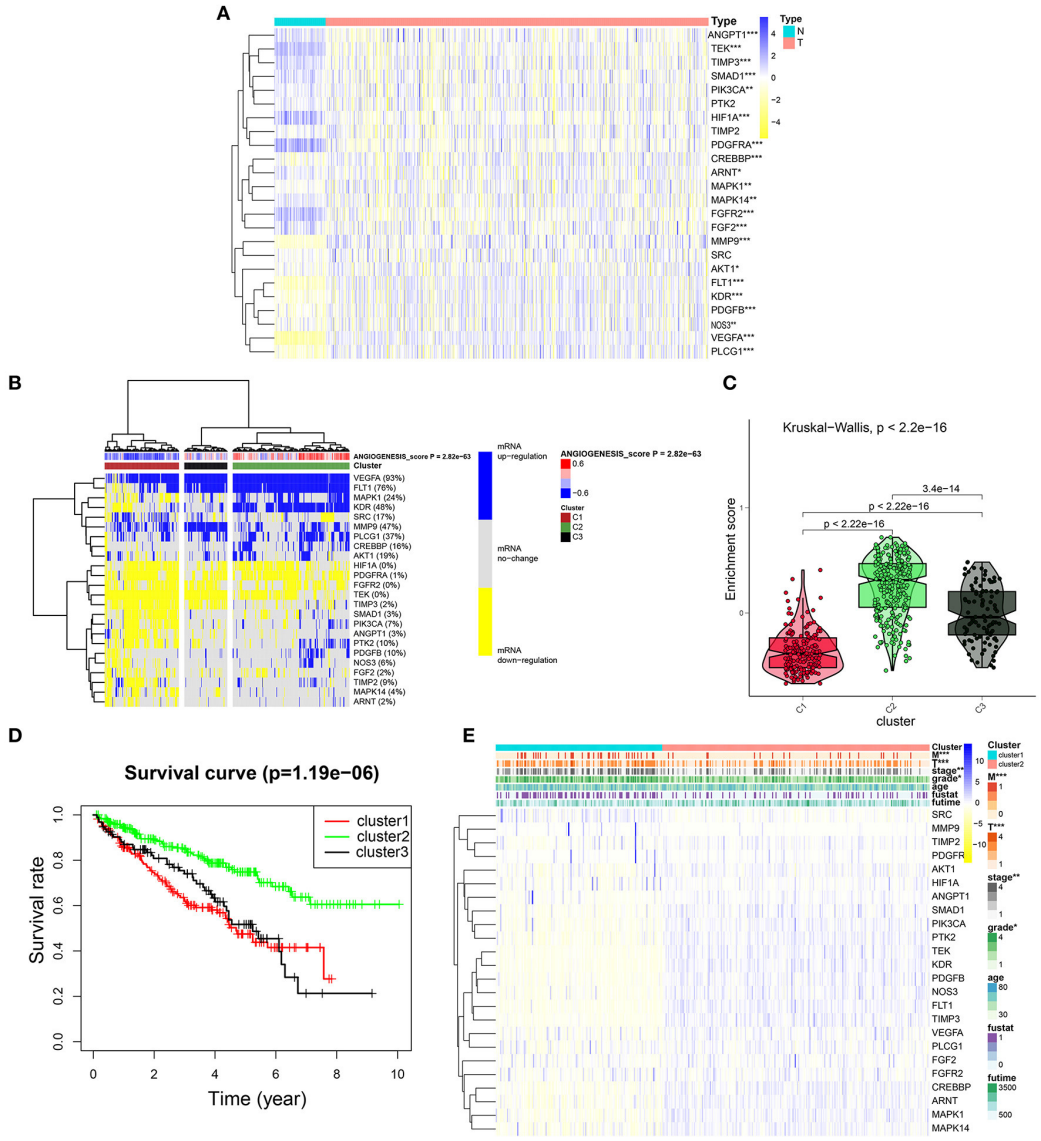
Cluster analysis based on the angiogenesis scores. **(A)** The heat map showed the expression levels of most of these genes were significantly different between the tumor tissues and normal tissues. In the color bar on the right side, blue represents gene up-regulation and yellow represents gene down-regulation. N (green) represents the normal sample, T (red) represents the tumor sample (*p < 0.05, **p < 0.01, ***p < 0.001). **(B)** Clustering of gene data from the TCGA database shows three clusters by the heat map: angiogenesis inactive (cluster 1 or C1), angiogenesis active (cluster 2 or C2), and normal angiogenesis (cluster 3 or C3). The percentage of patients whose genes are upregulated is given on the right side of the figure. In the color bar on the right side, blue represents mRNA upregulation, yellow represents mRNA downregulation, and gray represents mRNA no-regulation. The angiogenesis score is projected to four colors, negative values are displayed in blue, and positive values are displayed in red. **(C)** The violin plot drawn by the “ggpubr” package shows that the enrichment scores of the three clusters from high to low are cluster 2, cluster 3, and cluster 1. The statistical method used here is the “kruskal.test” and the *p*-values are displayed above the clusters. **(D)** The survival curve of the three clusters. The survival rate of cluster 2 is higher than cluster 1 and cluster 3. The red line represents cluster 1; the green line represents cluster 2; and the black line represents cluster 3. The abscissa represents the number of years and the ordinate represents the survival probability. **(E)** The heat map shows the correlation between the two clusters and the clinicopathological features. In the color bar on the right side, blue represents gene upregulation, and yellow represents gene downregulation (*p < 0.05, **p < 0.01, and ***p < 0.001).

To further explore the expression of angiogenesis-related genes in KIRC, we plotted their related heat maps (Figure 4A). We observed that the expression levels of most of these genes were significantly different between the tumor tissues and normal tissues. Next, a univariate Cox regression analysis of these genes in KIRC was carried out (Supplementary Figure 2, Supplementary Tables 9, 11), with the forest plot showing the HRs with 95% *CI*s and *p*-values. It was found that a high expression level of *TEK, MAKP1, PDGFB, PIK3CA, CREBBP, ANGPY1, FLT1, PTK2, NOS3, TIMP3, KDR*, and *ARNT* correlated with better survival rates in the patients with KIRC. By contrast, the high expression of *FGF2, SRC*, and *PDGFRA* correlated with the worse survival rates. Next, we clustered the angiogenesis-related genes obtained from the dataset of TCGA and divided the patients into three clusters based on the final angiogenesis score and gene expression level. C1 comprised the angiogenesis-inactive tumor tissues, C2 the angiogenesis-active tumor tissues, and C3 the tumor tissues with normal angiogenesis (Figure 4B, Supplementary Tables 6, 7). The violin plot clearly showed that the enrichment scores for the three clusters were in the order of C2 > C3 > C1 (Figure 4C). Next, we plotted the survival curves for the three clusters to determine whether the clustering was reasonable. The patients in C2 had significantly higher overall survival rates than the patients in C1 and C3 (Figure 4D), indicating that the angiogenesis score represented a protective factor. Finally, we explored the correlation between two of the clusters and the clinicopathological features (Figure 4E) and found that a higher angiogenesis score was negatively correlated with the tumor size (T), grade, and stage and tumor metastasis (M).

### Relationship Between the Drug Sensitivity and the Angiogenesis Clusters

**Figure 5:**
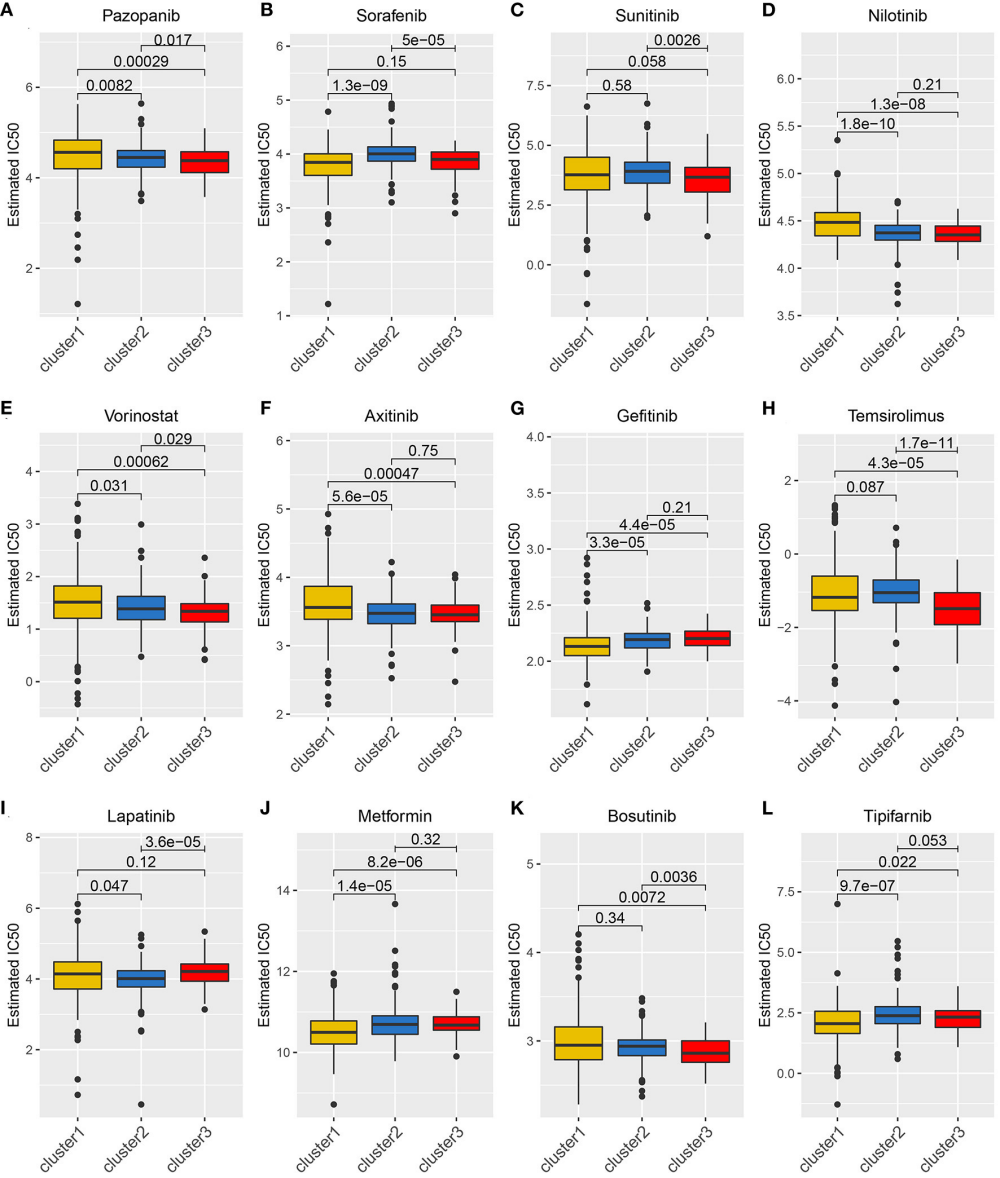
The relationship between drug sensitivity and the angiogenesis clusters. The box plots of the estimated IC50 for 12 types of common chemotherapeutic agents are shown in **(A–L)** for cluster 1 (yellow), cluster 2 (blue), and cluster 3 (red). The 12 types of chemotherapeutic agents are pazopanib, sorafenib, sunitinib, nilotinib, vorinostat, axitinib, gefitinib, temsirolimus, lapatinib, metformin, bosutinib, and tipifarnib. The *P*-values are displayed above the clusters and the *P* < 0.05 were considered statistically significant. The box plots showed drug sensitivities among the angiogenesis clusters were different.

Considering that molecularly targeted therapy is currently a common way to treat KIRC, we obtained related data from the GDSC database to assess the response of these three angiogenesis clusters to 12 types of drugs. These drugs primarily include both commonly used targeted therapeutics, especially for kidney cancer, and classical drugs in tumor research, such as metformin. For the treatment of recurrent or metastatic RCC, the targeted drug therapy is preferred. This includes the use of tyrosine kinase inhibitors (e.g., sunitinib, sorafenib, axitinib, and pazopanib) and mTOR inhibitors (e.g., Texiromu) (27). Metformin, an activator of AMP-activated protein kinase (AMPK), is primarily used for the management of type 2 diabetes. The studies have shown that metformin exerts antitumor effects against different types of cancer (e.g., breast, colon, liver, prostate, and kidney cancer) (28–30) primarily by reducing glycemia to cutoff the phosphoinositide 3-kinase (PI3K)/mitogen-activated protein kinase (MAPK) pathway or by activating the AMPK pathway (31). Therefore, it is necessary to explore the correlation between these targeted drugs and the angiogenesis pathway. Hence, we conducted a drug sensitivity analysis and obtained estimated IC_50_ values of the drugs for each sample. A lower IC_50_ value is indicative of better drug sensitivity. The ridge regression model revealed the different drug sensitivities among the angiogenesis clusters to be as follows: pazopanib: C3 > C2 > C1; sorafenib: C1 > C2; sunitinib: C3 > C2; nilotinib: C2 > C1; vorinostat: C3 > C2 > C1; axitinib: C2 > C1; gefitinib: C1 > C2; temsirolimus: C3 > C1; lapatinib: C2 > C1; metformin: C1 > C2; bosutinib: C3 > C2; and tipifarnib: C1 > C2 (Figures 5A–L).

### Correlations Between the Angiogenesis Score and the Classical Cancer-Related Genes or Immune Cell Infiltration

**Figure 6:**
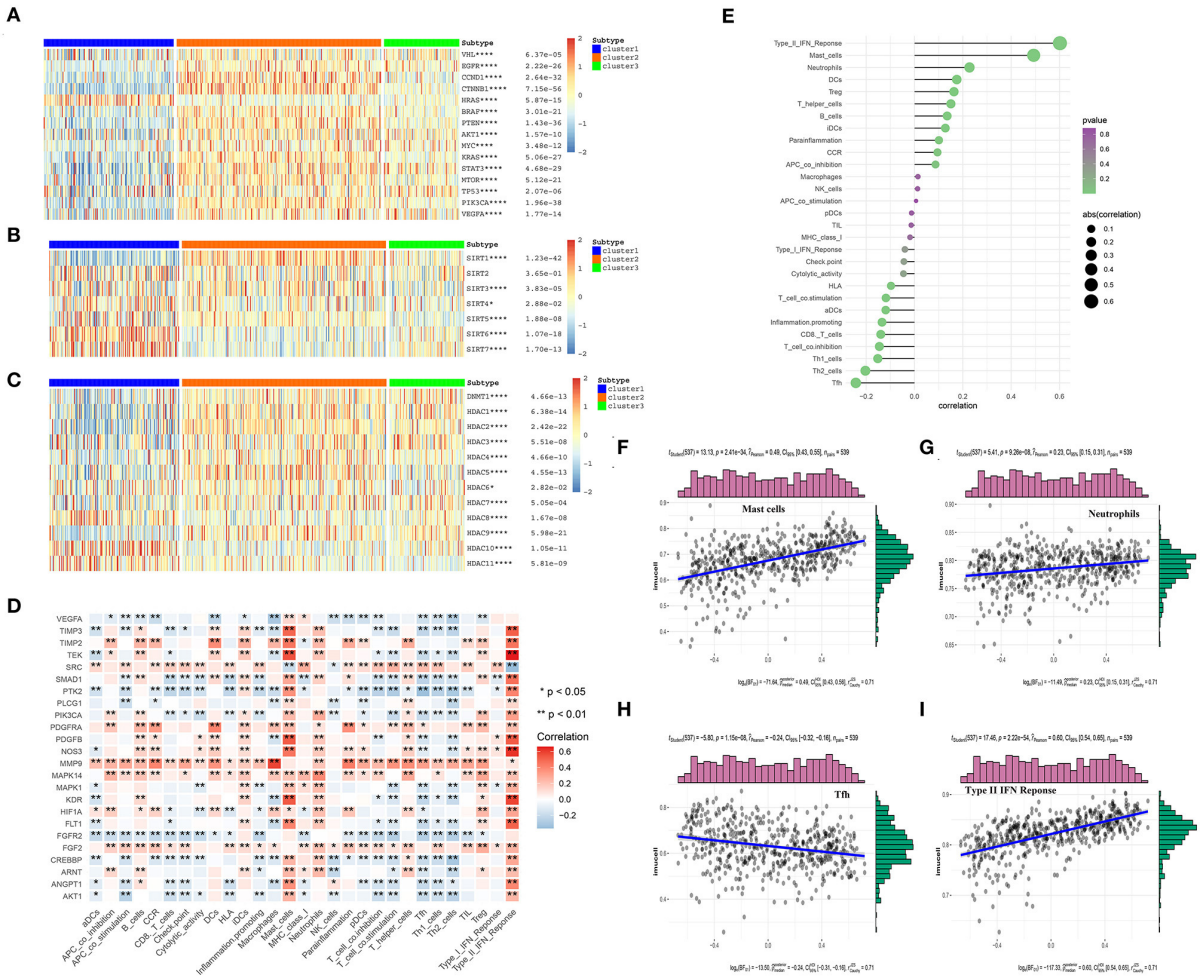
The correlations between the angiogenesis score and the classical cancer-related genes or immune cell infiltration. **(A–C)** Heatmap shows that the angiogenesis-score is correlated with other signaling pathways in KIRC. **(A)** The correlation with oncogenes and tumor suppressor genes. **(B)** The correlation with sirtuin family genes. **(C)** The correlation with histone deacetylases (HDAC) family genes. The statistical method used in A–C is “ANOVA” (*p < 0.05, **p < 0.01, ***p < 0.005, and ****p < 0.001). **(D)** The heat map shows that the correlation between the angiogenesis pathway genes and immune cells infiltration. The “ggplot2” and “dplyr” packages in R Studio were then used to draw the heat map, with Spearman's correlation coefficient being applied for the statistical analysis. Red represents positive correlation and blue represents negative correlation (*p < 0.05 and **p < 0.01). **(E)** The plot shows the correlation between the angiogenesis-score and immune cells infiltration. On the right side of the plot, the area of the sphere represents the degree of abs (correlation) and the color indicates the *p*-value. **(F–I)** The scatter diagram shows the correlation between the angiogenesis-score and four immune-infiltration-related substances. The angiogenesis-score was found to be positively correlated with the infiltration of type II IFN response cells, mast cells, and neutrophils, and negatively correlated with that of Tfh cells.

To further explore the differential expression patterns of the oncogenes and tumor suppressor genes in the three angiogenesis clusters, their related heat maps were plotted. We found that the expression levels of the oncogenes *CCND1, BRAF, AKT1, MYC, KRAS, MTOR, PIK3A*, and *VEGFA* were significantly higher in C2 than in C1. Additionally, the expression levels of the tumor suppressor genes von Hipel-Lindau (*VHL*), *TP53*, and *PTEN* were significantly lower in C1 than in C2 (Figure 6A). Among these tumor suppressor genes, the mutation of *VHL* can result in the overexpression of hypoxia-inducible factor-1 alpha (HIF-1α) protein, which is considered a hallmark of KIRC (32, 33). Continuously activated HIF-1α is shown to be related to cell proliferation, angiogenesis, and epithelial–mesenchymal transition, leading to the progression of KIRC and its metastasis to other organs (32, 34). The above results indicate that the poor prognosis of C1 may be correlated with the inhibition of tumor suppressor gene expression, which may play a more important role than the activation of oncogenes in C2. Additionally, the expression level of the oncogene *HRAS* was significantly higher in C1 than in C2.

In recent years, many studies have shown that SIRTs are involved in a variety of biological processes related to tumorigenesis, such as the changes in both the tumor-related metabolic pathways and tumor microenvironment and uncontrolled cell proliferation. In different cancer types and under different experimental conditions, SIRTs are thought to play a complex role as oncogenes or tumor suppressors (35). In our study, the level of *SIRT1* expression was significantly higher in the angiogenesis-active group than in the angiogenesis-inactive group. By contrast, the levels of *SIRT4, SIRT6*, and *SIRT7* expression were significantly lower in the angiogenesis-active group (Figure 6B). One study showed that *Patrinia scabiosaefolia* induced the death of 786-O cells *via* metabolic disruptions mediated by SIRT1 and mTOR signaling (36). Thus, SIRT1 inhibitors may be more effective for the patients who belong to the angiogenesis-active group. However, another study showed that SIRT1, SIRT3, and SIRT6 function as the tumor suppressors in RCC (37). In summary, these results indicate that SIRTs have a strong correlation with the angiogenesis signaling pathway and may act synergistically to promote or inhibit the multiple processes in the progression of KIRC.

Histone deacetylases, which catalyze the removal of acetyl groups from the histones and non-histone lysine residues, play an important role in gene transcription regulation (38) and are closely related to tumorigenesis and tumor metastasis. HDAC inhibition has recently become a clinically validated strategy for the treatment of cancer (39). We found that the expression levels of *HDAC1, HDAC2, HDAC3, HDAC4, HDAC5*, and *HDAC9* were significantly higher in the angiogenesis-active group than in the angiogenesis-inactive group. By contrast, the expression levels of *HDAC8, HDAC10*, and *HDAC11* were significantly lower in the angiogenesis-active group (Figure 6C). These results may offer new directions for the future precision treatment of tumors. For example, because *HDAC2* was almost exclusively expressed in the angiogenesis-active group, the use of HDAC2 inhibitors may be more beneficial for patients with an active angiogenesis pathway.

The tumor microenvironment is composed of immune cells, tumor cells, stromal cells, and secreted cytokines and chemokines (40) and regulates the occurrence and progression of cancer (41). As an important component of the tumor microenvironment, the immune cells are closely related to the clinical outcome of cancer and are effective targets for anticancer treatment (42). In addition, angiogenesis plays a key role in regulating the tumor immune microenvironment (43). To investigate the correlation between the angiogenesis pathway and immunity in the patients with KIRC, we performed a correlation analysis between the angiogenesis pathway and immune cell infiltration (Figure 6D) and found that many genes related to the angiogenesis signaling pathway were associated with the immune cell infiltration, especially *PDGFRA, MMP9, MAPK14, FGF2*, and *FGFR2*. Among these, *PDGFRA, MMP9, MAPK14*, and *FGF2* were positively correlated—whereas *FGFR2* was negatively correlated—with immune cell infiltration. Next, we analyzed the correlation of the angiogenesis score with immune cell infiltration (Figures 6E–I), whereupon it was found to be positively correlated with the infiltration of type II IFN response cells, mast cells, and neutrophils, and negatively correlated with that of Tfh cells.

### Construction of a Risk Model Using the LASSO–Cox Regression Analysis

**Figure 7:**
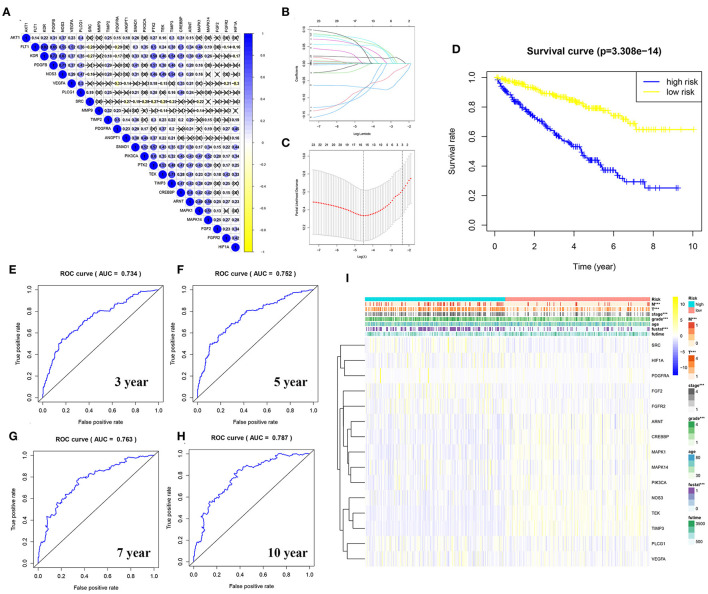
Construction of a risk model using the least absolute shrinkage and selection operator (LASSO)–Cox regression analysis. **(A)** The plot shows the result of co-expression relationships of 24 angiogenesis pathway genes in KIRC. In the color bar on the right side, blue represents positive correlation and yellow represents negative correlation. **(B)** The LASSO coefficient profiles of angiogenesis pathway genes in KIRC. **(C)** The distribution and median value of the risk scores. Fifteen genes were screened by LASSO Cox regression analysis. **(D)** The survival curve was obtained based on this model. The overall survival rates of patients in the high-risk group were significantly lower than those of the patients in the low-risk group. Blue represents the high-risk group and yellow represent the low-risk group. **(E–H)** ROC curve of 3-, 5-, 7-, and 10-years, area under the curve (AUC) of the curve are 0.734, 0.752, 0.763, and 0.787, respectively. **(I)** The correlation of risk score and the clinicopathological characteristics. The color bar shows the expression of genes. Yellow represents the upregulation of genes, blue represents the downregulation of genes (***p < 0.001).

To explore the relationship between the genes in the angiogenesis pathway, co-expression analysis of the 24 angiogenesis-related genes was carried out, and the results are shown in Figure 7A. To determine whether the angiogenesis-related genes could be used to establish a model for predicting the clinical outcomes of patients with KIRC, the LASSO–Cox regression analysis of the 24 genes was performed, and 15 were finally selected to build the risk score model (Figures 7B,C, Supplementary Tables 10, 12). The patients with KIRC were divided into a high-risk group and a low-risk group according to their RS values. The overall survival rates of patients in the high-risk group were significantly lower than those of patients in the low-risk group (Figure 7D, Supplementary Table 12). To explore the prognostic prediction efficiency of the new survival model in these same patients, a ROC curve analysis was performed. The areas under the ROC curves of the survival model for predicting 3-, 5-, 7-, and 10-year survival rates were 0.734, 0.752, 0.763, and 0.787, respectively, indicating that the risk model had a high predictive value (Figures 7E–H). Survival probability predicted by our angiogenesis prognostic signature was superior to the m6A-related lncRNA signature constructed by Yu et al. (25) and the seven-MDEG signature constructed by Hu et al. (26). For the patients with KIRC whose survival time was 1–4-years, our prognostic signature also showed a higher predictive accuracy compared with the autophagy-related long non-coding RNA signature constructed by Yu et al. (24). Additionally, for the patients whose survival time was 4–8-years, our prognostic signature showed similar predictive accuracy compared with the autophagy-related long non-coding RNA signature (24) (Supplementary Figure 3). A further statistical test was performed on the differences between the risk subgroups and a heat map was drawn to visualize the correlation between the RS and clinical data (Figure 7I). We found that the risk model was correlated to tumor metastasis (M) and the tumor size (T), stage, grade, and fustat, with patients in the high-risk group tending to have an advanced histological grade and to be in an advanced clinical stage.

### Predicting the Outcome of Patients With KIRC Using a Nomogram

**Figure 8:**
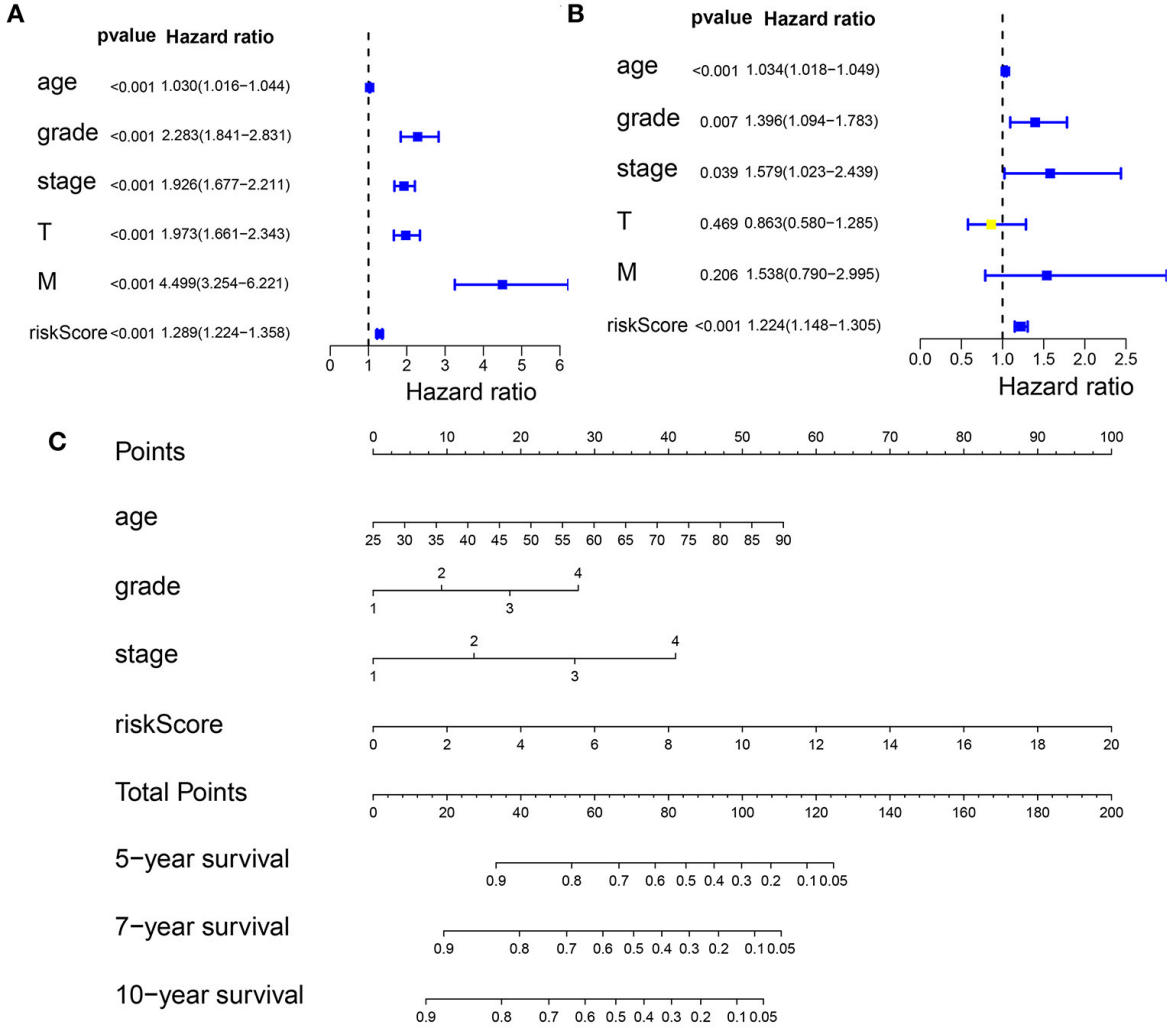
Predicting the outcome of patients with KIRC using a nomogram. **(A)** The univariate Cox regression analysis of the association among risk score (RS), the clinicopathological parameters [age, grade, stage, tumor size (T), and tumor metastasis (M)] and the overall survival (OS) of the patients with KIRC. The forest plot showed that the age, tumor grade, stage, T, M, and RS correlated with the OS of the patients (*p* < 0.05). **(B)** A multivariate Cox regression analysis of the association among the RS, clinicopathological parameters, and the OS of the patients with KIRC. The forest plot revealed that the patient's age, grade, stage, and RS were independent risk factors correlated with OS (*p* < 0.05). **(C)** Nomogram drawn by the “rms” package in R Studio incorporated riskScore, age, grade, and stage, which can be used to predict the outcome of patients with KIRC. The second to fifth lines represent the patient's age, tumor grade, tumor stage, and RS. The total score in the sixth row is the sum of the scores for each item from the second to fifth lines. The 5-, 7-, and 10-year survival rates were predicated based on the total score.

First, we performed a univariate Cox regression analysis on the RS and other clinicopathological features of the patients with KIRC (Figure 8A, Supplementary Table 13). The forest plot showed that the patient age, tumor grade, stage, and size (T), tumor metastasis (M), and RS correlated with the overall survival of the patients. The multivariate Cox regression analysis (Figure 8B, Supplementary Table 14) revealed that the patient age, tumor grade and stage, and RS were independent risk factors correlated with the overall survival. On the nomogram based on the risk model (Figure 8C), the second to ninth lines represent the patient age, tumor grade, tumor stage, RS, total points, 5-, 7-, and 10-year survival, respectively. The total score in the sixth row is the sum of the scores for each item from the second to fifth lines. The 5-, 7-, and 10-year survival rates were predicated based on the total score. For example, if the total score is 100, then the 5-year survival rate is approximately 0.3.

### TIMP3 Inhibition of Clear Cell RCC Growth *in vitro*

**Figure 9:**
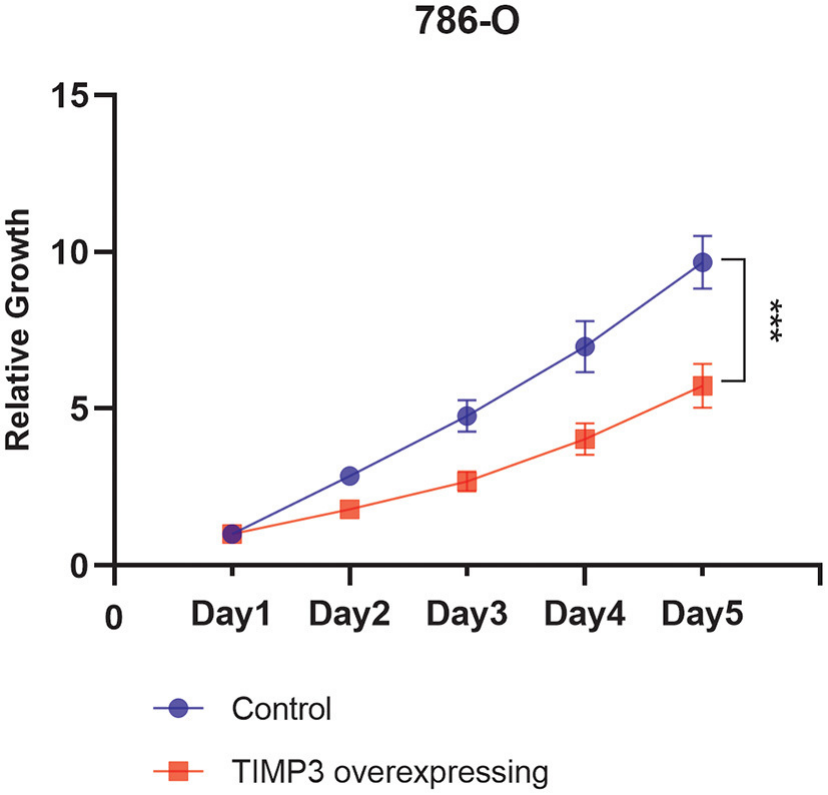
The proliferation curve of 786-O cells, which showed that the proliferation of tissue inhibitor of metalloproteinase 3 (TIMP3)-overexpressing 786-O renal cancer cells was significantly inhibited (***p < 0.001).

The viability of *TIMP3*-overexpressing 786-O renal cancer cells was determined using the CCK-8 assay, which showed that the proliferation of these cells was significantly inhibited (Figure 9). These results were consistent with our previous bioinformatics analysis, which indicated that *TIMP3* was a protective gene.

## Discussion

Angiogenesis, the mechanisms of which are only gradually beginning to be understood, refers to the formation of new blood vessels from the existing ones during organ development and wound healing (44). The VEGF family consists of six growth factors (VEGFA through VEGFF), which play the most critical role in angiogenesis by binding to receptors VEGFR1 through VEGFR3 and neuropilin (45). As a process that is highly regulated by the pro-angiogenic and anti-angiogenic factors, angiogenesis is disrupted and dysregulated in cancer (46). Tumor-driven hypoxia increases the expression of pro-angiogenic factors, leading to the formation of new blood vessels that are required for the growth of solid tumors (47). Until recently, the treatment with drugs targeting VEGF or the VEGFR pathway was the main approach for the advanced KIRC therapy.

Based on known research findings, we first chose to study the genetic mutations of angiogenesis pathway genes in 32 types of cancer. We then analyzed the alterations in the expression of the angiogenesis-related genes and explored whether they existed as protective genes or risk genes in the different types of cancer. We observed that most angiogenesis-related genes existed as protective genes in the patients with KIRC, which was inconsistent with the previous findings that overexpression of the angiogenesis pathway can lead to cancer development. It was previously shown that the activation of VEGF resulted in an improved blood supply and enriched source of nutrients for KIRC (48), whereas the inhibition of VEGFA inhibited the proliferation, promoted apoptosis, and suppressed the migration and invasion of 786-O KIRC cells (49). However, in our study, we found that *VEGFA* was neither a protective nor a risk gene. In another study, GLI1 and GLI2 were shown to be activated by the PI3K/protein kinase B (AKT) pathway, which was negatively correlated with the overall survival of KIRC patients (50). By contrast, our analysis showed that *PIK3CA* existed as a protective gene, an inconsistent phenomenon. The possible reason for these contradictory results is that there are still undiscovered pathways that may influence each other or simply that tumors are heterogeneous. Therefore, we focused our attention on the dataset of the patients with KIRC, dividing them into three clusters according to their angiogenesis scores and angiogenesis-related gene expression patterns. We found that the overall survival rates of patients in the angiogenesis-active cluster were significantly higher than those of the patients in the angiogenesis-inactive cluster, indicating once again that the genes involved in angiogenesis were mostly protective. However, the reason for this phenomenon remains unclear.

The agents targeting the angiogenesis pathway, such as sorafenib and sunitinib, are the mainstay therapeutics for metastatic KIRC (11, 12). Therefore, we looked at the roles of some of the most common drugs used to target the angiogenesis pathway genes in KIRC therapy. We found that the three patient clusters showed different sensitivities to the drugs studied, which suggests that a more personalized treatment plan could be provided to the patients based on their patterns of angiogenesis-related gene expression. For example, the use of pazopanib and nilotinib may be more beneficial to a patient whose angiogenesis pathway is highly active, whereas sorafenib and gefitinib may be more beneficial to a patient whose angiogenesis pathway is inactive.

The infiltrating inflammatory cells are an important part of the overall tumor mass. Initially, it was thought that these immune cells were part of the response of the host for combating the tumor; however, it was gradually recognized that most tumors were not regarded as foreign to the host and that inflammation/immune cell infiltration promoted the tumor growth and metastasis instead (51, 52). Angiogenesis plays an important role in immunosuppression and can lead to primary and secondary resistance to immune checkpoint inhibitors. VEGF and the growth factor angiopoietin-2 participate in this process by inhibiting the proliferation and differentiation of the activated immune effector cells while at the same time recruiting suppressive tumor-related immune cells (53). Therefore, in this study, we explored the correlation between the immune cell infiltration-related factors and angiogenesis pathway genes. We found that the mast cells, neutrophils, and Treg cells correlated positively with the angiogenesis score. The Treg cells, a subpopulation of CD4+ helper T cells, can facilitate the tumor progression by suppressing the antitumor immune responses of the host (54). In addition, the mast cells are also important contributors to immune-mediated tumor growth (51, 55). The neutrophils can play both tumor-promoting and antitumor functions, depending on their differentiation state and the presence of TGF-β (56). Therefore, the relationship of the angiogenesis score with the mast cells and Treg cells did not fit our expectations. The findings may be related to the complexity of the immune system and its interaction with the angiogenesis pathway. Given that the immune system of the host is considered to be the best tool for fighting cancer by limiting the spread of tumor cells, the development of immunotherapy has revolutionized the first-line treatment of metastatic KIRC (57, 58). The combination of pembrolizumab (a programmed cell death-1 inhibitor) and axitinib (a tyrosine kinase inhibitor) resulted in better outcomes than sunitinib among the patients with previously untreated advanced RCC (59).

Histone deacetylases are major epigenetic regulatory factors whose dysfunctional deacetylase activity is closely related to tumorigenesis and tumor metastasis (60). HDAC1 overexpression inhibits tumor suppressor p53 and VHL, but induces HIF-1a and VEGF, and increases angiogenesis. Conversely, HDAC inhibitors derepress p53 and VHL and repress HIF-1a and VEGF and correspondingly decrease the angiogenesis signaling. In addition to their direct antitumor effects, HDAC inhibitors have the ability to improve the recognition of tumors by immune cells, which may contribute to their antitumor activity indirectly (61). In this study, we found that most of the oncogenes, tumor suppressor genes, and HDACs were either positively or negatively correlated with the angiogenesis pathway. Therefore, the HDAC inhibitors offer a new strategy for tumor treatment, and our research results can further provide new directions for future precision tumor treatment. For example, the expression level of *HDAC1* in the angiogenesis-active group was significantly higher than that in the angiogenesis-inactive group, suggesting that the use of HDAC1 inhibitors may be more beneficial to the former group of patients.

Next, we constructed a risk model using LASSO–Cox regression analysis to predict the survival rate of patients with KIRC. The areas under the ROC curves indicated that this model has a high predictive value. Finally, we incorporated the RS, patient age, and tumor grade and stage into a nomogram for predicting the 5-, 7-, and 10-year survival rates of the patients with KIRC. Currently, other predictors of risk or survival based on different mechanisms or aspects do exist for KIRC. For example, Yu et al. constructed two prognostic signatures on the basis of autophagy-associated long non-coding RNAs (lncRNA) and m6A-related lncRNAs, respectively, both of which could effectively predict the outcome of patients with KIRC (24, 25). In another study, a 7-methylated differentially expressed gene signature was found to be a powerful prognostic factor for these patients (26). On the whole, our angiogenesis prognostic signatures exhibit a higher predictive accuracy for the patients with KIRC compared with the above prognostic signatures. Our current model adds to these other prognostic models and may provide more comprehensive and useful suggestions for the development of personalized therapies for patients with KIRC.

## Data Availability Statement

The original contributions presented in the study are included in the article/Supplementary Material, further inquiries can be directed to the corresponding author/s.

## Author Contributions

GW and QW conceived and designed this study and revised the manuscript. XC and WS performed the statistical analysis, data interpretation, and was a major contributor in writing the manuscript. XL and NL revised the manuscript. All the authors read and approved the final manuscript.

## Funding

This study was supported by the Scientific Research Fund of the Liaoning Provincial Education Department (no. LZ2020071), the Liaoning Province Doctoral Research Startup Fund Program (no. 209), and Dalian High-level Talent Innovation Support Plan (no. 2021-3-10).

## Conflict of Interest

The authors declare that the research was conducted in the absence of any commercial or financial relationships that could be construed as a potential conflict of interest.

## Publisher's Note

All claims expressed in this article are solely those of the authors and do not necessarily represent those of their affiliated organizations, or those of the publisher, the editors and the reviewers. Any product that may be evaluated in this article, or claim that may be made by its manufacturer, is not guaranteed or endorsed by the publisher.

## Supplementary Material

Supplementary Figure 1 **(A,B)** The copy number variation (CNV) frequency of the 24 angiogenesis pathway genes is shown for the 32 tumor types. **(C)** The single nucleotide variation (SNV) frequency of the 24 angiogenesis pathway genes is shown for the 32 tumor types. The color code bar on the right side refers to the frequency of CNV and SNV, red represents high frequency and yellow represents low frequency.

Supplementary Figure 2 The forest plot showed the hazard ratios (HRs) with 95% *CI* and the *P*-values of the angiogenesis-related genes.

Supplementary Figure 3 The area under the curve (AUC) values of the receiver operating characteristic (ROC) curve for the predictive performance of our signature compared with another three prognostic signatures.

Supplementary Table 1 Copy number variation (CNV) gain frequency of the angiogenesis pathway genes across 32 cancer types.

Supplementary Table 2 The CNV loss frequency of the angiogenesis pathway genes across 32 cancer types.

Supplementary Table 3 The single nucleotide variation (SNV) frequency of the angiogenesis pathway genes in 32 cancer types.

Supplementary Table 4 The LogFCs of the angiogenesis pathway genes across cancer types.

Supplementary Table 5 Hazard ratios of the angiogenesis pathway genes across cancer types.

Supplementary Table 6 The cluster and the angiogenesis score information.

Supplementary Table 7 The cluster and the angiogenesis score information.

Supplementary Table 8 The interactions among the different genes in the angiogenesis-related pathway.

Supplementary Table 9 The univariate Cox regression analysis on these angiogenesis-related genes in kidney renal clear cell carcinoma (KIRC).

Supplementary Table 10 The coefficient of selected angiogenesis-related genes.

Supplementary Table 11 The univariate Cox regression analysis on these angiogenesis-related genes in kidney renal clear cell carcinoma (KIRC).

Supplementary Table 12 The least absolute shrinkage and selection operator (LASSO) risk of the KIRC samples.

Supplementary Table 13 The univariate Cox regression analysis on the risk score and other clinicopathological features of the patients with KIRC.

Supplementary Table 14 The multivariate Cox regression analysis on the risk score and other clinicopathological features of the patients with KIRC.
