# Supplementary figures and images for: Angiogenesis Pathway in Kidney Renal Clear Cell Carcinoma and Its Prognostic Value for Cancer Risk Prediction
Source: Front Med (Lausanne). 2021 Oct 28;8:731214. doi: 10.3389/fmed.2021.731214 (PMC8581140; doi:10.3389/fmed.2021.731214)

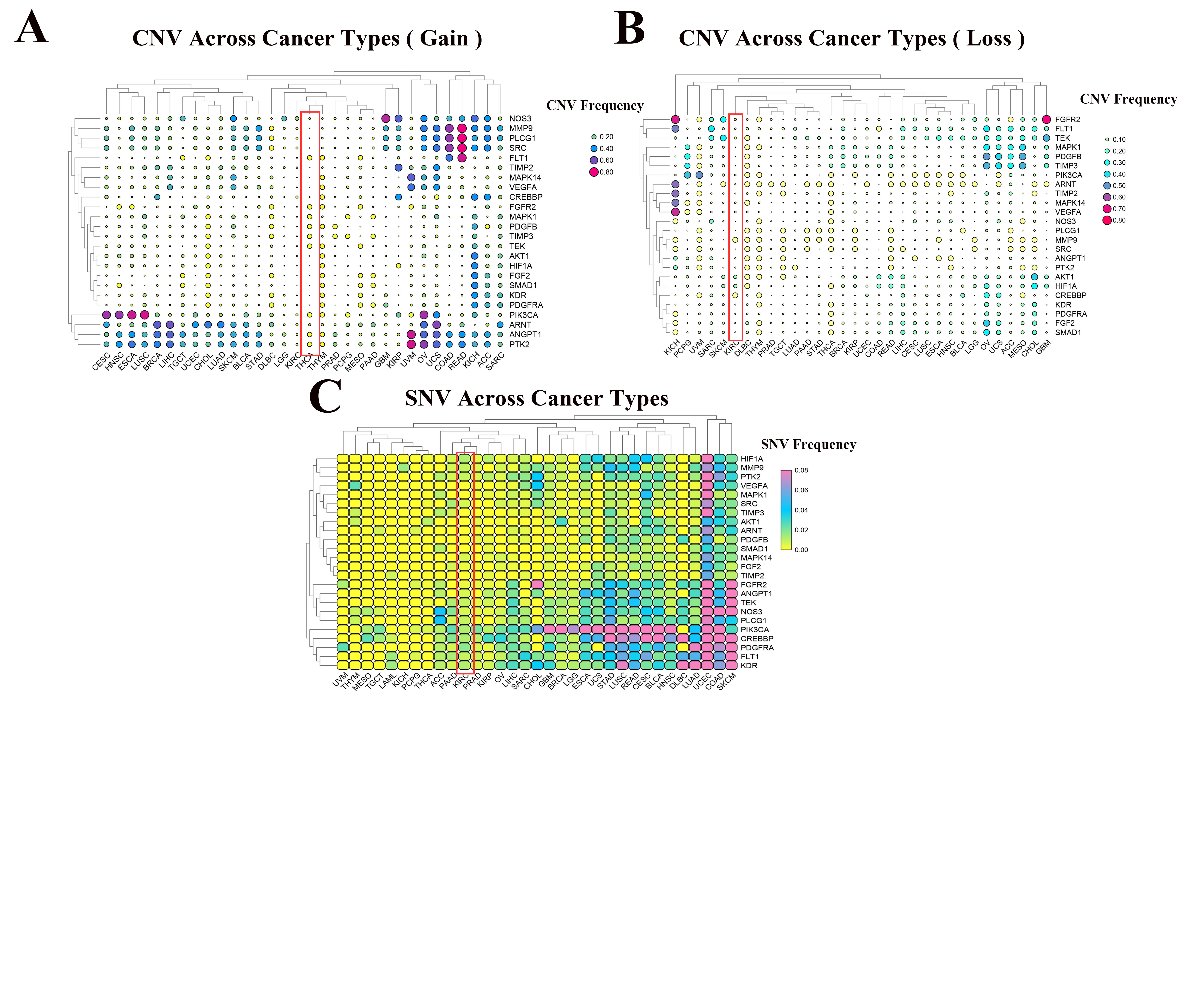

Supplement: Supplementary Figure 1 — (A,B) The copy number variation (CNV) frequency of the 24 angiogenesis pathway genes is shown for the 32 tumor types. (C) The single nucleotide variation (SNV) frequency of the 24 angiogenesis pathway genes is shown for the 32 tumor types. The color code bar on the right side refers to the frequency of CNV and SNV, red represents high frequency and yellow represents low frequency. [file Image_1.PNG]

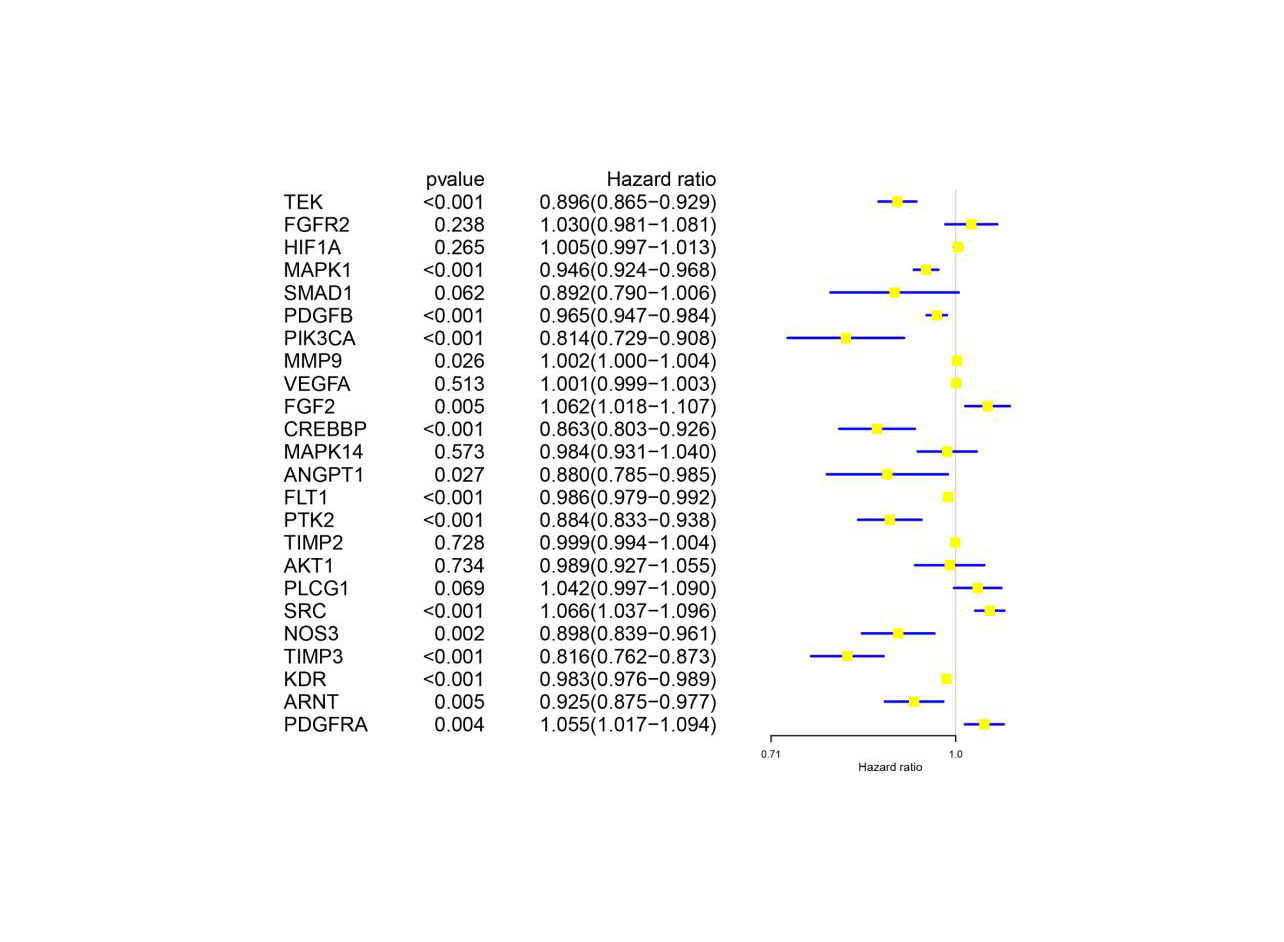

Supplement: Supplementary Figure 2 — The forest plot showed the hazard ratios (HRs) with 95% CI and the P-values of the angiogenesis-related genes. [file Image_2.tif]

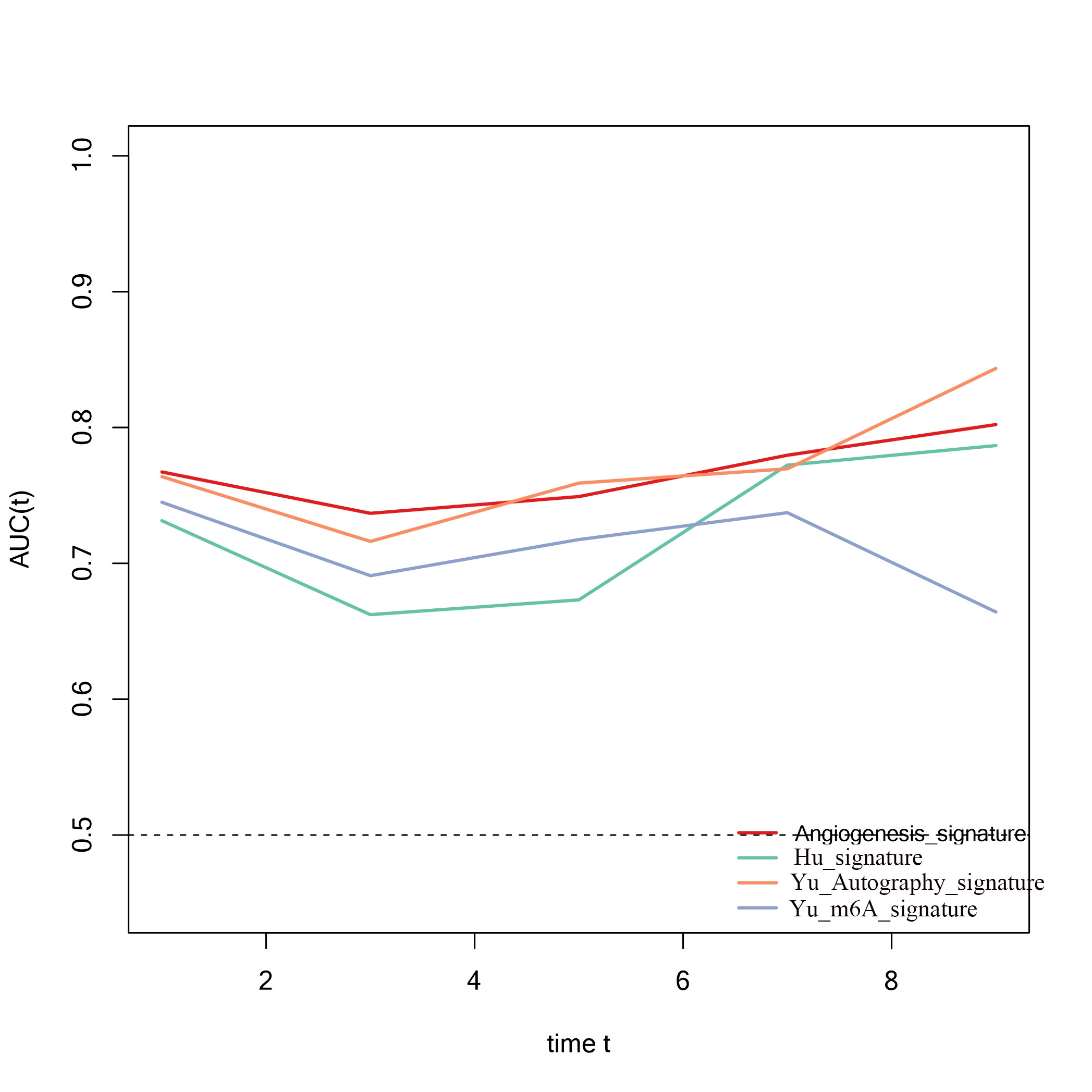

Supplement: Supplementary Figure 3 — The area under the curve (AUC) values of the receiver operating characteristic (ROC) curve for the predictive performance of our signature compared with another three prognostic signatures. [file Image_3.JPEG]
